# Exploring the memory of the gut microbiome: a multifaceted perspective

**DOI:** 10.3389/frmbi.2024.1363961

**Published:** 2024-04-25

**Authors:** Amine Zorgani, Bhaskar C. Das

**Affiliations:** ^1^ The Microbiome Mavericks, Lille, France; ^2^ Arnold & Marie Schwartz College of Pharmacy & Health Sciences, Long Island University – Brooklyn, Brooklyn, NY, United States

**Keywords:** microbiome, immunology, epigenetics, gut health and function, memory

## 1 Introduction

The gut microbiome, often regarded as a 'forgotten organ,' is an intricate ecosystem of microorganisms residing in the gastrointestinal tract (Brody, 2020). Its dynamic interplay with various bodily systems suggests a form of 'biological memory.' This memory concept is predicated on the microbiome's ability to respond and adapt to dietary (De Angelis et al., 2020; Bourdeau-Julien et al., 2023) and epigenetic changes (Kim, 2017), and the host’s immunity (Zheng et al., 2020). The potential for the microbiome to 'remember' past exposures and modify future responses could have far-reaching implications for our understanding of human health and disease.

The memory capability of the gut microbiome is also intricately tied to its accessibility and responsiveness to a wide array of environmental exposures over the course of a lifetime. Early microbial colonization in infancy, for instance, plays a crucial role in developing appropriate immune responses, metabolic pathways, and even behavioural traits (Jian et al., 2021; Donald and Finlay, 2023; Hunter et al., 2023). Disruptions to microbial composition during this critical window seem to have enduring effects. Rodent studies have shown that low diversity microbial colonization causes exaggerated inflammation and anxiety-like symptoms months after normalization of the microbiota (Bowerman et al., 2021). This implies immune and neural imprinting by pioneer gut microbes (Jin et al., 2021; Shevchenko et al., 2023).

The gut microbiome's phenotypic heterogeneity and genetic diversity are pivotal in its adaptive capabilities and ecological memory. Phenotypic variability, stemming from non-genetic factors, allows microbes to rapidly adapt to environmental changes through diverse responses (Ackermann, 2015). Concurrently, genetic diversity enriches functional capabilities enabling the microbiota to flexibly respond to dietary shifts and other changes (Heintz-Buschart and Wilmes, 2018). This interplay between phenotypic plasticity and genomic heterogeneity underpins the microbiome's resilience and its crucial role in host health.

This opinion piece aims to dissect the evidence and theories surrounding the memory-like characteristics of the gut microbiome, discussing how these traits could influence everything from metabolism to mental health, and how microbiome composition, phenotypes modulate physiology by genetical alternation.

## 2 Ecological memory of the gut microbiome

Diet is a primary factor influencing the gut microbiome’s composition and function. Studies have shown that dietary changes can lead to rapid shifts in microbial populations, indicating a responsive and adaptable microbial community. Hullar et al. (2014) documented how specific dietary components can select for certain microbial taxa, which in turn affect nutrient metabolism and even host behavior. This adaptability suggests a form of dietary memory, where past dietary patterns influence current microbial composition and function. Other concepts suggests that the microbiome’s dietary history might even modulate cognitive functions and mental health, indicating a complex interplay between diet, microbiome, and brain function (Li et al., 2009; Galland, 2014).

The gut microbiome exhibits an ‘ecological memory’ of past nutrient exposures, specifically carbohydrates. This memory is encoded rapidly, within a day of exposure to a nutrient like inulin (Letourneau et al., 2022). The strength of this memory correlates with the nutrient dose and persists for days, influencing the microbiome’s metabolic potential. The concept of ecological memory includes aspects like lag (the time before a differential response to stimuli), duration, and strength. For instance, a lag in response was observable by the second day of inulin treatment in an artificial gut model (Letourneau et al., 2022). This suggests that the gut microbiome can encode memory to a nutritional stimulus within a day​​.

The duration and strength of microbiome memory were assessed through various experiments, revealing that the microbiome’s potential to degrade inulin remained enhanced even when doses were separated by three days. Metabolomic analyses confirmed alterations in microbial activity and environment after repeated nutrient exposure, such as increases in short-chain fatty acids following the second inulin dose (Letourneau et al., 2022)​​. Investigations into specific ecological shifts showed that changes in transcription and abundance of primary degraders like *Bacteroides caccae* played a role in encoding memory to inulin exposure (Letourneau et al., 2022).

There are also indications that the gut microbiome remembers past infections, subsequently eliciting an increased resistance to infection with similar pathogens during later invasions (Nobs and Elinav, 2021). This process involves molecular mechanisms where a pathogenic insult by a strain of *Yersinia pseudotuberculosis* leads to compositional and functional changes in the gut microbiota, which then induce protection from subsequent exposure to the pathobiont *Klebsiella pneumoniae* (Nobs and Elinav, 2021). Notably, mice with a wild mouse microbiota, characterized by prior exposures to multiple infections, exhibited similar features to the “trained” microbiota of previously infected mice. These findings underline the microbiota’s ability to develop active resistance strategies against recurring pathogen. Mice infected with salmonella maintain elevated antibodies against the bacteria up to a year later compared to uninfected controls (Mittrücker and Kaufmann, 2000). This primed immune state is transmissible via faecal transplants from previously infected mice. The mechanisms likely involve tighter microbial-immune crosstalk after infection (Schuster et al., 2019).

A recent study demonstrated a new aspect of bacterial behavior, demonstrating a heritable form of memory in *Escherichia coli* (Bhattacharyya et al., 2023). The study discovered that *E. coli* can “remember” its swarming experiences over several generations, a capability hitherto largely unexplored in bacterial decision-making. This memory is encoded in the bacteria’s iron levels, which serve as a molecular basis for storing and recalling information about critical survival behaviors like swarming and biofilm formation. The study suggests that varying iron concentrations trigger distinct behavioral responses: low iron levels initiate a memory-driven, swift migratory swarm in search of iron, while high levels signal an environment favorable for biofilm development. This discovery not only challenges our understanding of bacterial adaptation and survival strategies but also opens new avenues in therapeutic interventions, targeting the iron-dependent memory mechanisms of bacteria.

Moreover, the enduring effects of antibiotics on the composition and functionality of the gut microbiome are well documented (Fishbein et al., 2023). Beyond acute damage, antibiotics seem to induce long-term changes to bacterial gene expression, metabolism, and virulence (Nel Van Zyl et al., 2022). This may explain observations that early antibiotic exposures can increase risk for certain metabolic and inflammatory diseases (Palleja et al., 2018). The notion is that disruptions to microbial memory imprints during development prime the immune system for hyper-reactivity later in life.

## 3 The immunological memory

The gut microbiome is a key player in the development and function of the immune system. It trains and modulates immune responses, a process that can be viewed as an immunological memory. Studies by Hullar et al. (2014) and Negi et al. (2019) highlight the gut microbiome’s role in shaping innate immune memory and the functional reprogramming of immune cells. This interaction is crucial for understanding how past microbial exposures can influence future immune responses. Additionally, Galland (2014) discusses the microbiome’s role in modulating systemic inflammation, which is implicated in conditions like chronic fatigue syndrome and fibromyalgia. This evidence suggests that the microbiome’s interaction with the immune system is not static but dynamic, capable of ‘remembering’ and responding to past encounters.

Pattern Recognition Receptors (PRRs) are a critical component of the innate immune system. They recognize specific molecular structures known as Microbe-Associated Molecular Patterns (MAMPs), which are expressed by a wide range of microbes, including those in the gut microbiome. This recognition triggers a series of signaling events that are essential for initiating an immune response (Kawai and Akira, 2010). The interaction between PRRs and MAMPs plays a pivotal role in the immune response, with implications for understanding the concept of memory in the gut microbiome.

First, PRRs, such as Toll-like receptors (TLRs), recognize MAMPs expressed by microbes, which is a crucial step in the innate immune response. This interaction enables the immune system to detect and respond to pathogenic invasions, triggering signaling cascades that initiate immune responses (Kawai and Akira, 2010). Following the recognition of MAMPs by PRRs, a series of signaling events occur. These events lead to epigenetic changes in immune cells, which do not involve permanent genetic alterations but result in sustained changes in gene expression and cell physiology. This epigenetic rewiring can be considered a form of cellular memory, impacting the activation and function of innate immune cells (Netea et al., 2016).

The concept of “trained immunity” has emerged from these interactions. It describes the capacity of innate immune cells, like macrophages, monocytes, and natural killer cells, to exhibit enhanced responsiveness upon reencountering pathogens. This property suggests a form of memory distinct from the traditional adaptive immune memory, grounded in the innate immune system’s ability to ‘remember’ past encounters with pathogens (Netea et al., 2016).

The gut microbiota plays a fundamental role in the induction, training, and function of the host immune system. The interactions between the gut microbiota and the host immune system, particularly through PRRs and MAMPs, are essential in maintaining the symbiotic relationship between the host and its diverse microbial population (Belkaid and Hand, 2014). The interaction between PRRs and MAMPs is crucial for understanding the mechanisms underlying microbiome memory. This interaction triggers signaling events and epigenetic modifications in innate immune cells, contributing to a memory-like response.

Building upon the foundational role of the gut microbiota in immune system development and function, the subsequent exploration of secreted Immunoglobulin A (IgA) introduces a nuanced layer to this complex interplay. IgA plays a pivotal role in the gut microbiome’s immunological memory, acting as a first line of defense in mucosal immunity (Pabst and Slack, 2020). IgA selectively binds to microbial antigens in the gut lumen, facilitating the maintenance of a balanced microbial community by promoting the clearance of pathogenic bacteria while sparing beneficial ones (Mantis et al., 2011; Huus et al., 2021). This selective mechanism underscores IgA’s critical role in shaping the gut microbiota composition, influencing host-microbial symbiosis and immune homeostasis. Studies have shown that IgA-coated bacteria are more likely to be excluded from the gut epithelial surface, preventing their overgrowth and potential pathogenicity (Viladomiu et al., 2017; Guo et al., 2021; DuPont et al., 2022).

Furthermore, IgA interacts with intestinal immune cells in various ways, influencing the immune response and contributing significantly to the gut’s ability to efficiently remember and respond to microbial encounters. This interaction involves several key mechanisms and cell types, including innate lymphoid cells (ILCs) and the adaptive immune system, particularly B cells and CD4+ regulatory T cells (Zheng et al., 2020).

ILCs, a heterogeneous population of innate immune cells, play a crucial role in secreting cytokines and chemokines rapidly to combat infection and promote mucosal tissue repair. These cells’ phenotypic diversity and functional plasticity are shaped by signals from the microbiome, with certain microbial metabolite sensors regulating their proliferation and function. For example, group 3 ILCs are crucial for host immunity and inflammation, mediating immune surveillance of microbiota configuration to facilitate early colonization resistance. This is done through the regulation of interleukin-22 (IL-22), demonstrating the intricate relationship between the gut microbiota and the immune system​​ (Castleman et al., 2019; Hepworth, 2023).

On the adaptive immune system front, B cells are essential mediators of gut homeostasis, producing a large array of secretory IgA antibodies responsive to commensals. Secretory IgA can be produced in both a T cell-independent and a T cell-dependent manner, with the latter playing a more significant role in shaping gut microbial communities (Tan et al., 2022). This mutualistic relationship between intestinal IgA and the microbiota contributes to maintaining a diversified and balanced microbiome, which in turn facilitates the expansion of Foxp3+ regulatory T cells. These cells sustain homeostatic IgA responses in a regulatory loop, highlighting the complex interplay between the immune system and the microbiota in the gut (Neumann et al., 2019)​​.

Additionally, IgA’s role extends beyond simple pathogen neutralization to include the modulation of microbial gene expression and metabolic activity. By binding to specific bacterial strains, IgA can influence bacterial behavior (Petersen et al., 2019), affecting nutrient acquisition (Petersen et al., 2019), virulence factor expression (Mathias and Corthésy, 2011), and biofilm formation (Woof and Russell, 2011). This interaction not only prevents colonization by pathogenic bacteria but also supports the establishment and maintenance of a beneficial microbial community essential for gut health. Research indicates that IgA-mediated selection of microbes can lead to the development of a microbiome that is more resilient to environmental perturbations, enhancing the host’s overall immune memory (Wilmore et al., 2018; Huus et al., 2020). The production of IgA is dynamically regulated by the gut’s immune system, reflecting past microbial exposures and shaping future immune responses (Moor et al., 2017; Donaldson et al., 2018). These insights into the immune system’s interactions with the gut microbiome can enhance our understanding of its role in health and disease.

## 4 Epigenetic regulation and memory

The relationship between the gut microbiome and the host’s epigenetic landscape is pivotal in understanding the microbiome’s memory-like characteristics. Epigenetics, involving heritable changes in gene expression without DNA sequence alteration, is significantly influenced by environmental factors, including gut microbial metabolites (Jaenisch and Bird, 2003).

The gut microbiota is known to produce various metabolites that modulate the host’s epigenetic machinery. Short-chain fatty acids (SCFAs) like butyrate, propionate, and acetate, produced from dietary fiber fermentation, are known to affect histone acetylation and methylation in host cells. Butyrate, in particular, is a known histone deacetylase (HDAC) inhibitor, affecting gene expression related to inflammation (Chriett et al., 2019). This activity facilitates histone acetylation, thus remodeling chromatin into an open and transcriptionally active state. This process can influence the expression of various genes, including those involved in metabolism and inflammation​​. For instance, in rat myotubes, butyrate supplementation led to histone hyperacetylation due to its HDAC inhibitory activity, affecting the expression of genes related to insulin resistance​​. Additionally, butyrate’s impact on gene transcription, particularly in metabolic control and its anti-inflammatory effects in human microvascular endothelial cells, further highlights its significant role in modifying gene expression (Chriett et al., 2019).

In the other hand, DNA methylation, a reversible and heritable form of gene expression regulation without altering the DNA sequence, has been observed in various bacteria, enabling them to swiftly adjust to changing environments (Sánchez-Romero and Casadesús, 2020). This epigenetic mechanism can significantly reduce the lag time of bacterial growth in response to encountered stimuli, allowing for a rapid shift in microbial community composition and functionality (Casadesús and Low, 2006; Shell et al., 2013).

The implications of DNA methylation in bacteria extend to the modulation of host-microbiome interactions, influencing not only microbial metabolism and growth but also the immune response of the host (Wang et al., 2020). Studies have shown that these epigenetic changes in gut microbiota can affect the expression of genes associated with host immunity and metabolism, thus contributing to a more nuanced understanding of the gut ecosystem’s dynamic nature (Wang et al., 2020). The ability of microbes to ‘remember’ and swiftly respond to environmental changes through mechanisms like DNA methylation underscores the complexity of microbial adaptation and its impact on health and disease.

## 5 Discussion

The concept of microbiome memory has significant implications for therapeutic interventions such as modulating the gut microbiota for clinical benefits, particularly in treating immune-related and neurological conditions (Letourneau et al., 2022) (Sherwin et al., 2018). Understanding the microbiome’s memory-like properties (both structurally and genetically) could also lead to novel immunotherapies and treatments for neurodevelopmental and neurodegenerative diseases (Kim, 2017; Negi et al., 2019). These therapeutic strategies might involve manipulating the microbiome to ‘reset’ or ‘retrain’ its memory, potentially offering new avenues for disease prevention and treatment.

The gut microbiome is deeply intertwined with host metabolism and plays a crucial role in drug metabolism, the efficacy and side effects of medications (Dodd and Cann, 2022). This interaction suggests that the microbiome’s history with certain drugs could impact future drug responses, a form of pharmacological memory.

The gut microbiota exhibits a preprogrammed nature, showing resistance to change despite various challenges. Letourneau et al. (2022) emphasizes this characteristic, suggesting that the microbiome is predisposed to maintain a certain balance, a form of ecological memory. This resistance to change could explain the microbiome’s ability to return to a baseline state after disturbances, influencing its response to environmental and dietary shifts.

The concept of memory in the gut microbiome, though metaphorical, underscores the microbiome’s dynamic and responsive nature. This memory-like feature suggests that past experiences of the microbiome can shape future responses, affecting host health and disease. Understanding this phenomenon offers promising avenues for therapeutic interventions, preventive healthcare, and provides a deeper insight into the intricate relationship between our gut microbiome and overall well-being. Embracing this concept will propel future research, leading to more personalized and effective health strategies.

## Author contributions

AZ: Conceptualization, Data curation, Investigation, Methodology, Writing – original draft, Writing – review & editing. BD: Writing – review & editing.
